# Delays in initial workflow cause delayed initiation of mechanical thrombectomy in patients with in-hospital ischemic stroke

**DOI:** 10.20407/fmj.2021-014

**Published:** 2021-11-25

**Authors:** Kenichiro Suyama, Shoji Matsumoto, Ichiro Nakahara, Yoshio Suyama, Jun Morioka, Akiko Hasebe, Jun Tanabe, Sadayoshi Watanabe, Kiyonori Kuwahara, Yuichi Hirose

**Affiliations:** 1 Department of Comprehensive Strokology, Fujita Health University, School of Medicine, Toyoake, Aichi, Japan; 2 Department of Neurosurgery, Fujita Health University, School of Medicine, Toyoake, Aichi, Japan

**Keywords:** In-hospital stroke, Mechanical thrombectomy, Treatment time intervals, Community-onset stroke

## Abstract

**Objectives:**

The benefit of mechanical thrombectomy for acute ischemic stroke is highly time dependent. However, time to treatment is longer for in-hospital stroke patients than community-onset stroke patients. This study aimed to clarify the cause of this difference.

**Methods:**

A retrospective single-center study was performed to analyze patients with large vessel occlusion who underwent mechanical thrombectomy between January 2017 and December 2019. Patients were divided into in-hospital stroke and community-onset stroke groups. Clinical characteristics and treatment time intervals were compared between groups.

**Results:**

One hundred four patients were analyzed: 17 with in-hospital stroke and 87 with community-onset stroke. Patient characteristics did not significantly differ between groups. Median door (stroke recognition)-to-computed tomography time (36 min vs. 14 min, P<0.01) and door-to-puncture time (135 min vs. 117 min, P=0.02) were significantly longer in the in-hospital stroke group than the community-onset stroke group. However, median computed tomography-to-puncture time (104 min vs. 104 min, P=0.47) and puncture-to-reperfusion time (53 min vs. 38 min, P=0.17) did not significantly differ.

**Conclusions:**

Longer door-to-puncture time in in-hospital stroke patients was mostly caused by longer door-to-computed tomography time, which is the initial part of the workflow. An in-hospital stroke protocol that places importance on early stroke specialist consultation and prompt transportation to the computed tomography scanner might hasten treatment and improve outcomes in patients with in-hospital stroke.

## Introduction

Multiple randomized trials have established mechanical thrombectomy as the standard of care for eligible patients with large vessel occlusion.^1–7^ Timely reperfusion is critical because earlier reperfusion improves functional outcomes: for every 30-minute delay, the absolute rate of a good outcome is reduced by 11%.^8–10^

Although in-hospital stroke (IHS) is much less common than community-onset stroke (COS), it is a significant problem. Approximately 6.5% to 15.0% of all strokes occur in patients already in the hospital.^11,12^ Previous reports have demonstrated significant delays in treatment and worse functional outcomes among patients with IHS.^13–17^ Furthermore, patients with IHS often cannot receive intravenous recombinant tissue plasminogen activator (rt-PA) because of comorbidities or contraindications.^13,18,19^ Therefore, mechanical thrombectomy plays a major role in many patients with IHS.

In this study, we aimed to clarify the cause of treatment delay in patients with IHS who underwent mechanical thrombectomy by comparing clinical features and treatment time intervals between IHS and COS patients.

## Methods

### Patient selection

This single-center retrospective study evaluated the angiographic and clinical data of 104 consecutive patients who underwent mechanical thrombectomy for large vessel occlusion stroke from January 2017 to December 2019. Large vessels were defined as the internal carotid artery, first or second segment of the middle cerebral artery, first segment of the posterior cerebral artery, basilar artery, and intracranial vertebral artery. IHS was defined as ischemic stroke occurring in a patient who had been hospitalized for more than 24 h.

Patients with acute ischemic stroke resulting from large vessel occlusion who were treated with mechanical thrombectomy were eligible for study inclusion. We excluded patients with re-occlusion after previous thrombectomy or large vessel occlusion during or after endovascular cerebral aneurysm or arteriovenous malformation treatment.

### IHS protocol

Our IHS protocol is outlined as follows: Once hospital staff identifies a patient with suspected stroke, they first contact the attending physician and stroke specialist. Both visit the patient as soon as possible to arrange and coordinate neuroimaging and determine if rt-PA and/or mechanical thrombectomy are indicated.

### Procedures

All procedures were performed using local anesthesia with a transfemoral approach. Intravenous heparin was administered and titrated to maintain an activated clotting time of two times the baseline value. A balloon guide catheter was inserted into the internal carotid artery or vertebral artery according to thrombus location. Mechanical thrombectomy was performed using a combined technique with both a stent retriever and aspiration catheter. Successful angiographic recanalization was defined as achieving thrombolysis in cerebral infarction (TICI) grade 2b or 3 flow.^20^

### Data collection and clinical outcomes

The following data were obtained and reviewed retrospectively from the medical records: patient age, sex, pre-morbid modified Rankin Scale (mRS) score, National Institutes of Health Stroke Scale (NIHSS) score on admission, location of the occluded vessel, and Alberta Stroke Programme Early Computed Tomography Score (ASPECTS).^21^ Stroke subtypes were classified according to the Trial of ORG 10172 in Acute Stroke Treatment criteria.^22^

Clinical outcome was evaluated using mRS score at discharge. Good outcome was defined as mRS score 0–2; poor outcome was defined as mRS score 3–6. Successful revascularization was defined as post-procedural TICI grade ≥2b. Adverse events were defined as procedure-related serious adverse events (arterial perforation, arterial dissection, subarachnoid hemorrhage, and vasospasm) and intracerebral hemorrhage. Symptomatic intracranial hemorrhage was defined based on the European-Australasian Acute Stroke Study II criteria as clinical deterioration likely caused by a hemorrhage that caused a NIHSS score increase ≥4 points.^23^

The following treatment time intervals were evaluated: (1) last well known (LWK) to door time (door time was considered stroke recognition time for patients with IHS), (2) door-to-computed tomography (CT) time, (3) door-to-needle time, (4) door-to-puncture time, (5) CT-to-puncture time, and (6) puncture-to-reperfusion time. For the IHS group, we also evaluated the time between stroke recognition and consultation with a stroke specialist.

This study was approved by the institutional ethics committee. The requirement for written informed consent for participation was waived due to the retrospective nature of the study. Patients were provided the opportunity to opt out of routine programmatic data analysis via the institutional website.

### Statistical analyses

Statistical analyses were performed using open source EZR software. Continuous data are presented as means with standard deviation or medians with interquartile range. Categorical data are presented as numbers with percentage. Statistical comparisons were conducted using the Student’s *t* test, Mann–Whitney U test, or Fisher’s exact test as appropriate. P<0.05 was considered significant.

## Results

During the study period, 112 patients with large vessel occlusion stroke underwent mechanical thrombectomy in our institution. Eight patients with re-occlusion after previous thrombectomy or large vessel occlusion after endovascular cerebral aneurysm or arteriovenous malformation treatment were excluded. Therefore, 104 patients were included for analysis: 17 with IHS and 87 with COS. Patient characteristics are presented in Table 1.

The IHS and COS groups did not significantly differ in terms of age, sex, pre-morbid mRS score, NIHSS score on admission, ASPECTS, anticoagulant medication, nor occluded vessel. The percentage of patients who received rt-PA was significantly lower in the IHS group than the COS group (12% vs. 51%, P<0.01). Approximately 85% of IHS patients who were recognized within 4.5 h of the LWK time had a contraindication to rt-PA. The most common contraindication was recent invasive treatment.

Cardioembolism was the most common ischemic stroke subtype in both IHS and COS patients. However, the distribution of subtypes differed between groups. The rate of other specific etiologies was significantly higher in patients with IHS than in patients with COS (24% vs. 14%, P<0.01). In the IHS group, the other specific-etiologies (n=4) included after lobectomy for lung cancer (n=3) and Trousseau syndrome (n=1). The detailed characteristics of the patients with IHS are illustrated in Table 2. The most frequent initial admission department was pulmonology (n=5), followed by the strokology (n=4) and cardiology (n=3) wards. The most common reason for admission was malignancy (n=8), followed by stroke (n=4).

**Figure 1:**
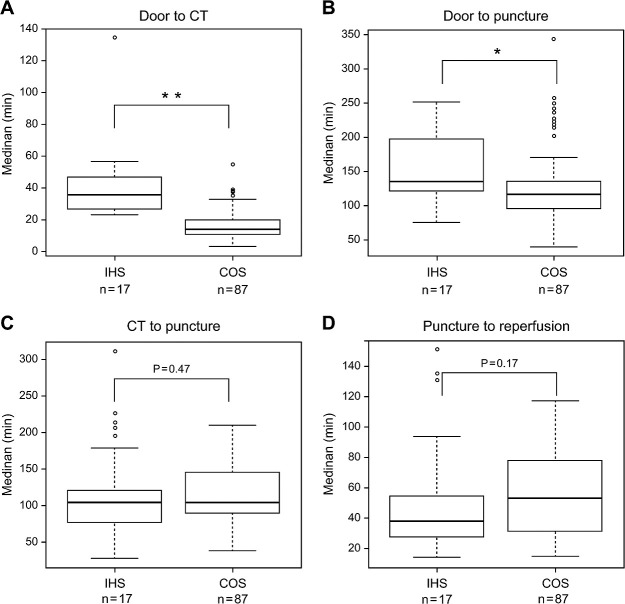
Comparison of treatment time intervals between the IHS and COS groups. Box-and-whisker plots of the time from (A) door to computed tomography (CT), (B) door to puncture, (C) CT to puncture, and (D) puncture to reperfusion. The horizontal line within the box represents the median and the top and bottom edges of each box indicate the interquartile range. Door-to-CT and door-to-puncture times were significantly longer in the IHS group than the COS group. * Significant difference with P<0.05. ** Significant difference with P<0.01. COS, community-onset stroke; IHS, in-hospital stroke; LWK, last well known

The time intervals from stroke recognition to treatment are depicted in Figure 1. The median LWK-to-door time (door time was considered stroke recognition time for patients with IHS) was shorter in the IHS group than the COS group but the difference was not significant (60 min vs. 80 min, P=0.23). The median door-to-CT time was significantly longer in the IHS group than the COS group (36 min vs. 14 min, P<0.01; Figure 1a). The median door-to-needle time was longer in the IHS group than the COS group but the difference was not significant (119 min vs. 63 min, P=0.23). The median door-to-puncture time was also significantly longer in the IHS group than the COS group (135 min vs. 117 min, P=0.02; Figure 1b). However, the times from median CT to puncture (104 min vs. 104 min, P=0.47), puncture to reperfusion (53 min vs. 38 min, P=0.17), and LWK to reperfusion (299 min vs. 274 min, P=0.55) did not significantly differ (Figure 1c, d). The median time from stroke recognition to stroke specialist consultation was 27 min. Neuroimaging was performed after consultation in five patients.

Treatment outcomes are presented in Table 3. The rates of successful recanalization (94% vs. 87%, P=0.69), adverse events (47% vs. 36%, P=0.42), and symptomatic intracranial hemorrhage (12% vs. 7%, P=0.62) did not significantly differ between the IHS and COS groups. Good outcome (mRS score 0–2) at discharge was achieved in six IHS patients (50%) and 26 COS patients (51%). Mortality did not significantly differ between the groups.

## Discussion

In the present study, we observed that door-to-puncture time was longer in IHS patients than COS patients. The main reason for this was significantly longer door-to-CT time, which is the initial part of the workflow. This phenomenon might be related to delayed consultation with the stroke specialist. The median time from recognition of symptoms to consultation with the stroke specialist was 27 min, and neuroimaging was performed after the consultation in some cases. Simultaneous consultations with the stroke specialist and attending physician are required as per our hospital protocol, although in actuality, consultation with the stroke specialist is often delayed. This delay can result in longer door-to-CT time. Previous studies have reported that delayed consultation with stroke specialists causes treatment delays in patients with IHS.^12,17,19,24^

Moreover, several studies have reported improvements in assessment and treatment times after establishing an IHS protocol.^25–28^ Three strategies were commonly employed in these studies: (1) implementing an IHS protocol, (2) repeated stroke education for key staff members, and (3) involving nurses with specialized stroke education in the initial response. Although our hospital had implemented an IHS protocol, stroke education for staff was insufficient and the initial response to IHS was limited to stroke specialists. These factors may have contributed to delayed treatment of IHS in our hospital. In the future, we plan to modify our IHS protocol algorithm to include stroke specialists and stroke-educated nurses in the initial stroke response. In addition, we plan to initiate a stroke education program for key staff members.

Several time intervals measured between stroke recognition and treatment in our study were longer in the IHS group than the COS group; however, outcomes did not significantly differ. This is because shorter LWK-to-door time compensated for the delays in neuroimaging and treatment in the IHS group. A well-designed IHS protocol that places importance on early consultation and prompt transportation to the CT scanner may lead to better outcomes in patients with IHS.

Thirteen patients in the present study experienced in-hospital large vessel occlusion that was recognized within 4.5 h. Among these, 11 (85%) patients had contraindications to rt-PA. Previous studies have also reported low utilization of rt-PA in IHS patients because of their comorbidities and contraindications.^19,24,29^ Therefore, mechanical thrombectomy plays a more important role in IHS patients than COS patients. We suggest that IHS protocols should include the use of mechanical thrombectomy in addition to rt-PA.

IHS patients have different baseline characteristics than COS patients.^29,30^ In this study, approximately half of the IHS patients had an underlying malignancy and the most frequent reason for hospital admission was surgery or treatment for malignancy. Moreover, there were four patients with IHS with other specific-etiologies of ischemic stroke subtypes, and the causes of three of them occurred after lobectomy for lung cancer. Cerebral embolism occurs in 0.2%–1.2% of patients who undergo lung cancer surgery; it typically develops following left upper lobectomy in patients with a long pulmonary vein stump.^31–33^ Patients who are at high risk of ischemic stroke after lung cancer surgery should be identified before surgery and closely monitored neurologically afterward.

Our institution has approximately 1500 beds and a comprehensive stroke center that accepts stroke patients at all times. The distance to the CT and angiography rooms is long and transportation and communication can be difficult because of our hospital’s large size compared with community hospitals. This might explain the long CT-to-puncture times observed among our study patients in both the IHS and COS groups.

This study had several limitations, including its single-center retrospective design and relatively small sample size. In addition, all patients had large vessel occlusion stroke and were treated using thrombectomy alone. Patients with other types of stroke and those who received other treatment were not examined. Future prospective studies that include all cases of IHS and assess workflow times in detail are warranted.

## Conclusions

Workflow times for mechanical thrombectomy were longer in IHS patients than COS patients in our institution. Delayed door-to-puncture time was mostly caused by delayed door-to-CT time, which is the initial part of the workflow. A well-designed IHS protocol that improves the initial part of the workflow might hasten treatment and improve outcomes in IHS patients.

## Figures and Tables

**Table1:** Characteristics of patients with in-hospital stroke and community-onset stroke

	IHS (n=17)	COS (n=87)	P-value
Age	76.6±8.3	74.6±12.8	0.53
Male	12 (70.6)	45 (51.7)	0.19
mRS score before onset	0 (0–2.0)	0 (0–1.5)	0.26
NIHSS score	12 (7–18)	20 (14–25)	0.06
ASPECTS	9 (7–10)	9 (8–10)	0.58
Hypertension	15 (88.2)	69 (79.3)	0.52
Dyslipidemia	8 (47.1)	40 (46.0)	1
Diabetes mellitus	5 (29.4)	21 (24.1)	0.76
Coronary artery disease	5 (29.4)	10 (11.5)	0.07
Smoking habit	13 (76.5)	49 (56.3)	0.18
Atrial fibrillation	7 (41.2)	56 (64.4)	0.10
Anticoagulation	5 (29.4)	18 (20.7)	0.52
rt-PA	2 (11.8)	44 (50.6)	<0.01
Site of occlusion			
Internal carotid artery	5 (29.4)	22 (25.2)	0.77
M1	5 (29.4)	41 (47.1)	0.20
M2	5 (29.4)	18 (20.7)	0.52
Posterior circulation	2 (11.8)	6 (6.9)	0.61
Ischemic stroke subtype			
cardioembolism	9 (52.9)	64 (73.6)	0.14
atherosclerosis	1 (5.9)	10 (11.5)	0.69
other specific-etiologies	4 (23.5)	1 (13.8)	<0.01
undetermined etiology	3 (17.6)	12 (13.8)	0.71

Data are presented as means±standard deviation, medians with interquartile range, or numbers with percentage. ASPECTS, Alberta Stroke Programme Early Computed Tomography Score; COS, community-onset stroke; IHS, in-hospital stroke; mRS, modified Rankin Scale; NIHSS, National Institutes of Health Stroke Scale; rt-PA, recombinant tissue plasminogen activator; M1, first segment of middle cerebral artery; M2, second segment of middle cerebral artery

**Table2:** Characteristics of patients with in-hospital stroke

	IHS (n=17)
Patient department at time of stroke	
Pulmonology	5 (29.4)
Strokology	4 (23.5)
Cardiology	3 (17.6)
Gastroenterology	2 (11.8)
Urology	2 (11.8)
Emergency	1 (5.9)
Reason for admission	
Surgery or treatment for malignancy	8 (47.1)
Lung	5 (29.4)
Prostate gland	1 (5.9)
Bladder	1 (5.9)
Liver	1 (5.9)
Stroke	4 (23.5)
Active cardiovascular disease	2 (11.8)
Surgical procedure	2 (11.8)
Infection	1 (5.9)

**Table3:** Comparison of treatment outcomes between the in-hospital stroke and community-onset stroke groups

	IHS (n=17)	COS (n=87)	P-value
Total number of passes	2 (1–3)	1 (1–2.5)	0.37
Successful recanalization	16 (94.1)	76 (87.4)	0.69
Adverse events	8 (47.1)	31 (36.0)	0.42
Symptomatic ICH	2 (11.8)	6 (7.0)	0.62
Good outcome at discharge	6 (35.3)	27 (31.0)	0.78
mRS score at discharge	4 (2–5)	4 (2–5)	0.55
Mortality	4 (23.5)	13 (14.9)	0.47

Data are presented as medians with interquartile range or numbers with percentage. COS, community-onset stroke; ICH, intracranial hemorrhage; HIS, in-hospital stroke; mRS, modified Rankin Scale
